# Exploring the Spectrum of Visual Illusions and Other Minor Hallucinations in Patients with Parkinson’s Disease in Lithuania

**DOI:** 10.3390/medicina60040606

**Published:** 2024-04-06

**Authors:** Neringa Jucevičiūtė, Renata Balnytė, Ovidijus Laucius

**Affiliations:** Department of Neurology, Medical Academy, Lithuanian University of Health Sciences, A. Mickeviciaus Str. 9, LT-44307 Kaunas, Lithuania

**Keywords:** Parkinson’s disease, visual illusion, minor hallucination, psychosis

## Abstract

*Background and Objectives*: Parkinson’s disease (PD) is associated with various non-motor symptoms, including minor hallucinations, comprising visual illusions and presence and passage hallucinations. Despite their occurrence, even in newly diagnosed PD patients, data regarding the prevalence and characteristics of minor hallucinations, visual illusions in particular, remain limited. The aim of this study was to address this knowledge gap by assessing the prevalence of minor hallucinations in PD patients, with a focus on visual illusions. *Materials and Methods*: In this prospective pilot study, we enrolled 35 PD patients without dementia and 35 age- and gender-matched PD-unaffected individuals. Cognitive function was assessed using the Montreal Cognitive Assessment, clinical data were collected, and all subjects were assessed via questionnaires regarding 20 types of visual illusions and other minor hallucinations. *Results*: The prevalence of minor hallucinations was significantly higher among PD patients compared to controls (45.7% vs. 11.4%, *p* = 0.003). PD patients reported visual illusions and presence hallucinations more frequently than the controls (37.1% vs. 8.6% and 22.9% vs. 2.9%, *p* = 0.009 and *p* = 0.028, respectively), with no significant difference in passage hallucinations (20% vs. 8.6%, *p* = 0.306). In the PD group, the most frequently observed visual illusions were complex visual illusions, kinetopsia, and pelopsia; the latter was also the most common visual illusion in the control group. PD patients experiencing visual illusions were more likely to report presence hallucinations compared to patients without visual illusions (53.8% vs. 4.5%, *p* = 0.002); no significant differences in other clinical characteristics were found. *Conclusions*: Minor hallucinations are a common phenomenon among PD patients without dementia, with a higher prevalence than among healthy controls. Visual illusions are the most prevalent type of minor hallucinations, affecting more than a third of PD patients, with complex visual illusions, kinetopsia, and pelopsia being the most frequently reported types.

## 1. Introduction

Psychotic manifestations affect up to 50–70% of individuals with Parkinson’s disease (PD) at some stage in their disease course and may be profoundly disruptive, contributing to increased mortality and morbidity, as well as caregiver distress [1]. Parkinsons’s Disease Psychosis (PDP), rather than motor dysfunction, stands out as the single greatest risk factor for nursing home placement among PD patients [2,3]; however, there are no universally accepted diagnostic criteria of PDP and, according to DSM-5, in most cases, PD psychotic symptoms are placed under the category of “Psychotic Disorder Due to Another Medical Condition”, requiring prominent hallucinations or delusions with episodes that cause significant impairment to the patient [4,5]. However, PDP presents a distinct pattern of psychotic symptoms, as recognized in 2007 with the proposal of diagnostic PDP criteria, emphasizing certain characteristic symptoms lasting for at least one month: hallucinations, delusions, illusions, and a false sense of presence [6]. Visual hallucinations are abnormal visual perceptions without a visual physical stimulus, in contrast to visual illusions which are misperceptions of real visual stimuli, and they are often referred to as “minor hallucinations” alongside presence hallucinations (a false sensation that another person is present nearby when nobody is actually there; also known as a false sense of presence) and passage hallucinations (fleeting, vague images in the peripheral vision) [6]. However, minor hallucinations are known to occur even in de novo PD patients, with one study showing a prevalence of 42% among 50 patients [7]. Since minor hallucinations can occur so early in the disease course, their inclusion in the provisional diagnostic PDP criteria is controversial, as acknowledged by the authors, who emphasized the necessity of documentation of these phenomena for their refinement in the future [6]. Nevertheless, minor hallucinations and, in particular, visual illusions remain highly underexplored. Due to a tendency of most studies to focus only on a limited subset of visual illusions despite their diverse range, their prevalence in different studies is extremely variable, ranging from 20% to 75% [8,9]. Notably, the latter prevalence was reported by, to our knowledge, the most comprehensive study on visual illusions in PD, which assessed the prevalence of 20 types of visual illusions in 40 PD patients, with some illusions experienced by multiple participants and others reported only once in the cohort [9]. In order to increase the understanding regarding minor psychotic phenomena in PD, we aimed to assess the prevalence of minor hallucinations in our Lithuanian cohort of PD patients and healthy controls, with a particular focus on the 20 types of distinct visual illusions described by Sasaki et al. [9]. 

## 2. Materials and Methods

### 2.1. Study Design and Participants

This prospective pilot study, conducted at the Hospital of Lithuanian University of Health Sciences Kaunas Clinics, included 35 PD patients and 35 age- and gender-matched PD-unaffected individuals. Patients diagnosed with PD were eligible for inclusion if they met all of the following criteria: (1) PD diagnosed based on the Movement Disorder Society’s clinical diagnostic criteria; (2) onset of the first motor symptoms of PD at age ≥ 50 years; (3) not treated with deep brain stimulation or lesional procedures (e.g., thalamotomy, pallidotomy). Subjects were excluded if they met any of the following criteria: (1) a history of central nervous system disease (i.e., epilepsy, stroke, multiple sclerosis); (2) psychiatric illness that could present with hallucinations (e.g., schizophrenia, schizoaffective disorder); (3) significant visual impairment (e.g., due to advanced glaucoma, age-related macular degeneration, or untreated severe cataract); (4) a previous diagnosis of dementia or Montreal Cognitive Assessment (MoCA) score < 21 points. Control subjects were eligible for inclusion if they did not have PD and did not meet any of the specified exclusion criteria. 

### 2.2. Background Motor and Cognitive Assessments

Medical information was gathered directly from the study participants, as well as from their electronic health records. Cognitive function was evaluated using the Lithuanian version of the MoCA (7.1). For each PD patient, the levodopa equivalent daily dose (LEDD) was calculated, and the disease severity was assessed with the modified Hoehn and Yahr scale [10]. 

### 2.3. Assessment of Visual Illusions and Other Minor Hallucinations

All participants were given a detailed explanation of the differences between hallucinations and visual illusions. Subsequently, they were asked a series of Yes/No questions for 20 types of visual illusions, visual hallucinations, presence hallucinations, and passage hallucinations. This study adopted the definitions of various visual illusions that is outlined in the work by Sasaki et al. [9]. Standardized examples were provided to participants when clarification was needed for a particular question. Both the definitions and the examples of phenomena are presented in Table 1. Participants who affirmed experiencing a phenomenon were asked to elaborate on specific details, ensuring accurate comprehension of the experience in question.

### 2.4. Statistical Analysis of Data

Statistical analyses were conducted using the SPSS software package (version 29.0; IBM). Descriptive statistics were calculated, and the normality of data was evaluated using histograms and the Shapiro–Wilk test. Given that all continuous variables were non-normally distributed, the Mann–Whitney U test was used to evaluate their differences between the groups. Fisher’s exact test was used for categorical variable comparison. Continuous variables are reported as median (interquartile range [IQR]), and categorical variables are reported as count (percentage). All *p* values are two-sided, and those that are inferior to 0.05 are considered statistically significant.

## 3. Results

There were no significant differences in clinical characteristics between the PD and control groups (Table 2). The majority of PD patients exhibited bilateral involvement without severe disability, with 32 (91%) individuals falling within the modified Hoehn and Yahr stages of 2.0 to 3.0.

**Figure 1:**
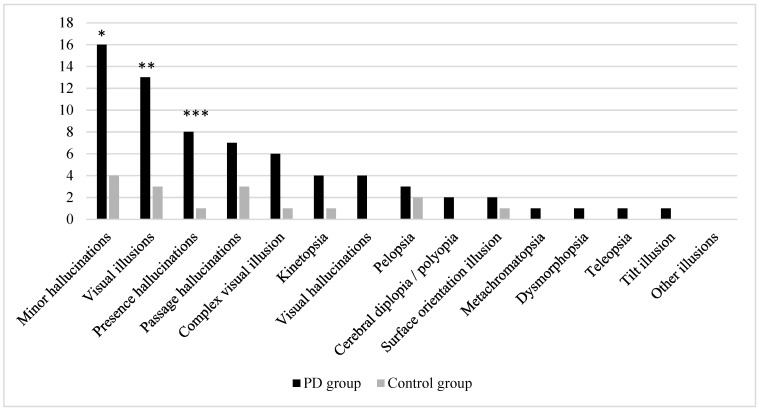
Frequency of reported visual illusions, other minor hallucinations, and visual hallucinations among study participants. Significant at: * *p* = 0.003, ** *p* = 0.009, and *** *p* = 0.028; in other cases, no statistically significant differences were found.

In total, 16 PD patients and 4 control subjects experienced at least one minor hallucination (45.7% vs. 11.4%, *p* = 0.003). Among these, the PD patients reported both visual illusions and presence hallucinations more frequently than the control subjects (37.1% and 22.9% vs. 8.6% and 2.9%, *p* = 0.009 and *p* = 0.028, respectively); however, despite passage hallucinations being more than twice as common in the PD group (20% vs. 8.6%), no statistically significant difference was observed in their occurrence (*p* = 0.306) (Figure 1).

Among the 16 participants who reported visual illusions, 9 (56.3%) experienced only one type of visual illusion. In the PD group, the most frequently reported visual illusions were complex visual illusions (*n* = 6), kinetopsia (*n* = 4), and pelopsia (*n* = 3), while in the control group, pelopsia was the most frequently reported visual illusion (*n* = 2). In total, nine types of visual illusions were not experienced by any participant (i.e., textural illusion, macropsia, micropsia, akinetopsia, Zeitraffer and Zeitlupen phenomena, upside-down illusion, and both types of visual perseveration). Polyopia and cerebral diplopia were grouped together into polyopia/cerebral diplopia, since both subjects who reported this type of visual illusion were not able to provide responses concerning the circumstances in which the illusion was experienced. Complex visual illusions, kinetopsia, and visual hallucinations were observed more than twice as frequently in the PD group; however when comparing the presence of these specific visual phenomena or other particular types of visual illusions, no statistically significant differences were found between the PD and control groups (Figure 1). 

Those PD patients who reported visual illusions tended to have lower total MOCA scores, a longer disease duration, and a higher LEDD; however, these differences were not statistically significant (Table 3). Notably, more than half of the PD patients with visual illusions also experienced presence hallucinations, whereas among those who did not report visual illusions, only one participant reported this phenomenon (53.8% vs. 4.5%, *p* = 0.002).

## 4. Discussion

In this study, we investigated minor hallucinations and their subtypes in PD patients without dementia. Our findings revealed a significantly higher prevalence of minor hallucinations and visual illusions in individuals with PD than in PD-unaffected individuals, with 46% of PD patients experiencing minor hallucinations and 37% experiencing visual illusions, of which the most frequently reported were complex visual illusions, kinetopsia, and pelopsia. Moreover, we did not find any significant association between the presence of visual illusions and clinical characteristics in the PD group, with the exception of a higher co-occurrence of presence hallucinations. 

The prevalence of minor hallucinations among PD patients varies significantly across different studies, and our results fell in the middle of this spectrum: one study found a prevalence of 39% among 262 PD patients [11], while another one showed that 42% of de novo untreated PD patients already reported minor hallucinations [7]. These figures might even underestimate the true prevalence, as certain studies have detected surprisingly high rates of visual illusions, a key component of minor hallucinations, with figures reaching as high as 75% [9]. Minor hallucinations have also been described in the healthy population, with a prevalence of 5% reported in one study [7]. Our observed prevalence of 11% among control subjects was slightly higher; however, this variation may be due to a small sample size. 

The 37% occurrence of visual illusions among PD patients in our study also falls within a range of prevalence rates that has been previously reported: 20% in a Lithuanian study [8], 43% found by Nishio et al. [12], and the notably higher 75% reported by Sasaki et al. [9]. Regarding the subtypes of visual illusions, our observed high prevalences of kinetopsia and complex visual illusions were consistent with the trends reported in other studies [8,12], but this observation was in contrast to the observations of Sasaki et al. [9], who identified dysmorphopsia as the predominant visual illusion, present in 35% of patients, followed by complex visual illusions. Notably, dysmorphopsia was reported by only one PD patient in our study and was absent in all 30 PD patients in another Lithuanian study [8]. All these differences may be partly attributed to methodological variations: the distinct phrasing of questions across languages, the different numbers of visual illusion types studied, e.g., only eight types in two of the studies [8,12], and variations in the stringency of cognitive criteria, e.g., 10% of PD patients who reported visual illusions in Sasaki et al.’s study had MoCA < 21 points [9]. In addition, cultural influences on the susceptibility to visual illusions have been observed, potentially arising from task design bias or underlying neurobiological mechanisms [13]. This could also account for some of the discrepancies in the prevalence and common subtypes of visual illusions found in our study and in another conducted in Lithuania, compared to the two Japanese studies mentioned [8,9,12]. Importantly, in our cohort, none of the PD patients who experienced minor hallucinations or visual hallucinations had disclosed their symptoms to their neurologist, and none had received a diagnosis of PDP. This could be attributed to a number of possible factors, including a stigmatized perception of these phenomena, a lack of awareness among patients, or the absence of structured evaluations by neurologists.

Despite a lack of statistical significance, we observed trends towards lower cognitive function, longer disease duration, higher LEDD, and a higher prevalence of dopamine agonist use in PD patients who reported visual illusions, which is in line with the findings of other studies [9,11,14]. However, there are no good data to suggest a direct causal relationship between these factors and minor hallucinations. The high prevalence of minor hallucinations in de novo PD patients without any dopaminergic medications indicates that these phenomena are part of PD itself from very early in the disease course [7]. The pathophysiological mechanisms behind psychotic phenomena in PD remain incompletely understood; nevertheless, some hypotheses have been proposed. Independently of disease duration, PD patients with visual hallucinations have greater both cortical and subcortical atrophy compared to PD patients with visual illusions [15]. This neurodegeneration may lead to “top-down” dysfunction, characterized by a lack of suppression of internally generated imagery, which, coupled with a decreased strength of external visual inputs („bottom-up“ dysfunction), could contribute to hallucinations; in contrast, visual illusions may stem from predominantly dysfunctional visual input without significant “top-down” dysfunction [16,17]. The “bottom-up” dysfunction is likely attributed to retinal changes that are directly associated with PD. The retina contains a specific subset of dopaminergic neurons that are crucial for enhancing sensory processing of visual information, which is affected in Parkinson’s disease, as demonstrated by optic coherence tomography, revealing retinal thinning in PD patients with visual hallucinations, but according to one study, not in those with visual illusions [15,16,18]. Retinal thinning could result from a primary ocular process or as a consequence of retrograde trans-synaptic degeneration triggered by cerebral changes [16]. In addition, cortical dysfunction, as shown by hypometabolism in the visual spatial processing supporting the temporo-parietal cortices, is also associated with both visual illusions and hallucinations in PD [12]. Other minor hallucinations, i.e., passage and presence hallucinations, could be linked to dysfunctional motion perception and eye movement control in PD [16]. Furthermore, it has been suggested that certain phenotypic subtypes of PD may have a higher occurrence of minor hallucinations, as supported by a study that found a greater incidence of minor hallucinations, particularly visual illusions, in patients with the postural instability gait difficulty phenotype compared with the tremor-dominant phenotype; however, it is important to note that this finding could have been influenced by confounding factors, since the postural instability gait difficulty phenotype was also associated with more severe motor and other non-motor symptoms, as well as higher LEDD [19]. Regarding the clinical course, insufficient data are available to state that minor hallucinations independently increase the risk of developing visual hallucinations; while some studies suggest a close association between these phenomena, showing that minor hallucinations (including visual illusions) usually precede visual hallucinations and may even represent their milder form, others have shown that only a small proportion of PD patients with visual illusions evolve towards visual hallucinations over 2 years [20,21]. Functional magnetic resonance imaging studies have suggested that dorsal attention network dysfunction may be a key factor in the progression from minor hallucinations to well-structured visual hallucinations [22]. Minor hallucinations could even be an early clinical marker of increased neurodegeneration. Newly diagnosed PD patients who develop minor hallucinations within the first 5 years after diagnosis have been found to have more extensive gray matter volume loss at baseline and an increased rate of atrophy during the first 2 years [23]. However, further studies are needed for definite conclusions; if they were to establish that minor hallucinations are a risk factor for accelerated disease progression, cognitive deterioration, or disabling symptoms in PDD, the early screening for minor hallucinations could prove very useful for clinical trials of potential disease-modifying therapies or drugs for PDP [20]. Given the diverse range and different prevalence of specific types of visual illusions in PD, future studies should focus on developing rapid and standardized questionnaires for screening of visual illusions and other minor hallucinations. Such screening tools are also required to increase our understanding of the neurobiological processes behind these phenomena. Furthermore, as had already been mentioned by the group which proposed the provisional diagnostic criteria of PDP, the documentation of “minor” psychotic symptoms is crucial for the refinement of PDP diagnostic criteria [6], and this will be complicated until we know which specific questions to ask patients about the experience of minor hallucinations. 

The majority of previous studies concentrated on only a select few types of visual illusions; therefore, this study represents only the second one assessing 20 types of visual illusions in PD patients. By also incorporating other minor hallucinations, this study pioneers in offering a thorough examination of the phenomenology of all minor hallucinations in PD. Additionally, comprehensive inclusion/exclusion criteria were applied to ensure the elimination of potential confounding factors, ensuring the determination of prevalence as specifically as possible within the context of PD. 

The present study has several limitations to be noted. Firstly, due to a small sample size, we did not identify all the visual illusions that have previously been described in other studies, and our prevalence estimates may be inaccurate. Secondly, this was a single-center study with only one examiner assessing each participant, limiting the generalizability of our findings. Thirdly, we did not perform a detailed ophthalmological examination. In addition, the test–retest reliability of our questionnaire was not evaluated. Lastly, the cognitive function assessment relied solely on MoCA rather than a detailed neuropsychological assessment, and therefore, it cannot be completely ruled out that some participants may have had mild PD dementia. 

## 5. Conclusions

In conclusion, our findings highlight the common occurrence of minor hallucinations among PD patients without dementia, with a higher prevalence than among healthy controls. This observation is particularly notable for visual illusions, the most prevalent type of minor hallucinations, affecting more than a third of PD patients, with complex visual illusions and kinetopsia being the most frequently reported types. Moreover, further studies focusing on the development of rapid and standardized questionnaires for the screening of visual illusions and other minor hallucinations are imperative for an increased understanding of psychotic-spectrum symptoms in PD and for the refinement of PDP diagnostic criteria.

## Figures and Tables

**Table 1:** Definitions and examples of various visual illusions, other minor hallucinations, and visual hallucinations, adapted from Sasaki et al. [9].

Phenomenon	Definition	Example
Metachromatopsia	The color of an object seems different	A blue cup seems red
Textural illusion	The surface of an object seems different	A flat table seems wavy
Dysmorphopsia	The shape of an object seems different	A straight candle seems distorted or bent in two
Macropsia	An object seems bigger in size	A cherry seems as big as an apple
Micropsia	An object seems smaller in size	An apple seems as small as a cherry
Teleopsia	An object seems farther than in reality	A table within arm’s reach seems a few meters away
Pelopsia	An object seems nearer than in reality	A table a few meters away seems within arm’s reach
Kinetopsia	A stationary object seems to be in motion	A stationary lamp seems to be moving, while other objects stay still
Akinetopsia	A moving object seems to be still	A person walking in front seems to suddenly stop, even though he is still moving
Zeitraffer phenomenon	An object seems to be moving faster	A slowly walking person seems to be running
Zeitlupen phenomenon	An object seems to be moving slower	A rapidly moving dog seems to be slowly walking
Tilt illusion	An object or the visual scene seems tilted	A candle on a table seems to be tilted by 45 degrees
Upside-down illusion	An object or the visual scene seems inverted	A clock seems inverted
Polyopia/cerebral diplopia	A single object appears as multiple (≥2), while other objects do not change in count; classified as cerebral diplopia if the number of objects “increases” upon continuous viewing	One painting appears as three paintings, while the number of other objects remains constant
Visual perseveration	Ongoing perception of an object after it has moved out of the visual field; classified as immediate perseveration/palinopsia (after a few minutes)/hallucinatory palinopsia (after a few days or later)	A person “re-appears” at the door after leaving the house a few minutes ago
Complex visual illusion	One object seems like another kind of object	A tree outside the window seems to be a person
Surface orientation illusion	False perception of surface orientation	A flat street seems to be going downhill
Passage hallucinations	Fleeting, vague images in the peripheral vision	A shadow of a dog is seen in the corner of the eye but disappears upon closer look
Presence hallucinations	False sensation that another person is present nearby when nobody is actually there	The sensation that another person is present behind one’s back when there is no one else in the room
Visual hallucinations	Abnormal visual perceptions without a physical stimulus	Despite the table being empty, there seems to be a book on it

**Table 2:** Clinical characteristics of PD patients and control subjects.

Clinical Characteristics	PD Group (*n* = 35)	Control Group (*n* = 35)
Sex, female	17 (48.6%)	17 (48.6%)
Age, years	67.0 (63.0–71.0)	67.0 (62.0–75.0)
Education, years	14.0 (12.0–16.0)	13.0 (12.0–18.0)
MoCA, points	23.0 (21.0–25.0)	24.0 (22.0–27.0)
Disease duration, years	8.0 (3.0–12.0)	NA
Modified Hoehn and Yahr stage	2.5 (2.0–3.0)	NA
LEDD, mg	700 (400–1150)	NA

Abbreviations: LEDD, levodopa equivalent daily dose; MoCA, Montreal Cognitive Assessment; PD, Parkinson‘s disease.

**Table 3:** Comparison of clinical characteristics between PD patients who reported visual illusions and those who did not.

	PD with Visual Illusions (*n* = 13)	PD without Visual Illusions (*n* = 22)	*p* Value
Sex, female	5 (38.5%)	12 (54.5%)	0.489
Age, years	67.0 (61.0–70.5)	67.5 (63.0–72.25)	0.719
Education, years	15.0 (12.5–16.5)	14.0 (12.0–16.0)	0.262
MoCA, points	22.0 (21.0–25.0)	23.5 (21.0–26.0)	0.201
Disease duration, years	10.0 (2.8–13.0)	7.0 (3.75–11.0)	0.504
Hoehn and Yahr stage	2.5 (2.0–3.0)	2.0 (2.0–3.0)	0.507
LEDD, mg	1003 (350.0–1457.0)	670.5 (468.8–856.3)	0.290
History of dopamine agonist use	10 (76.9%)	15 (68.2%)	0.709
History of amantadine use	7 (53.8%)	12 (54.5%)	1
History of trihexyphenidyl use	1 (7.7%)	1 (4.5%)	1
Visual hallucinations reported	3 (23.1%)	1 (4.5%)	0.134
Passage hallucinations reported	4 (30.8%)	3 (18.2%)	0.383
Presence hallucinations reported	7 (53.8%)	1 (4.5%)	0.002

Abbreviations: LEDD, levodopa equivalent daily dose; MoCA, Montreal Cognitive Assessment; PD, Parkinson‘s disease.

## Data Availability

Data are contained within the article.
